# Supplementary material for: Nitric Oxide Distribution Correlates with Intraluminal Thrombus in Abdominal Aortic Aneurysm: A Computational Study
Source: Bioengineering (Basel). 2025 Feb 17;12(2):191. doi: 10.3390/bioengineering12020191 (PMC11851545; doi:10.3390/bioengineering12020191)
Supplement: Supplementary file 1 [file bioengineering-12-00191-s001.zip › bioengineering-3459177-supplementary.pdf]

Supplementary Materials.

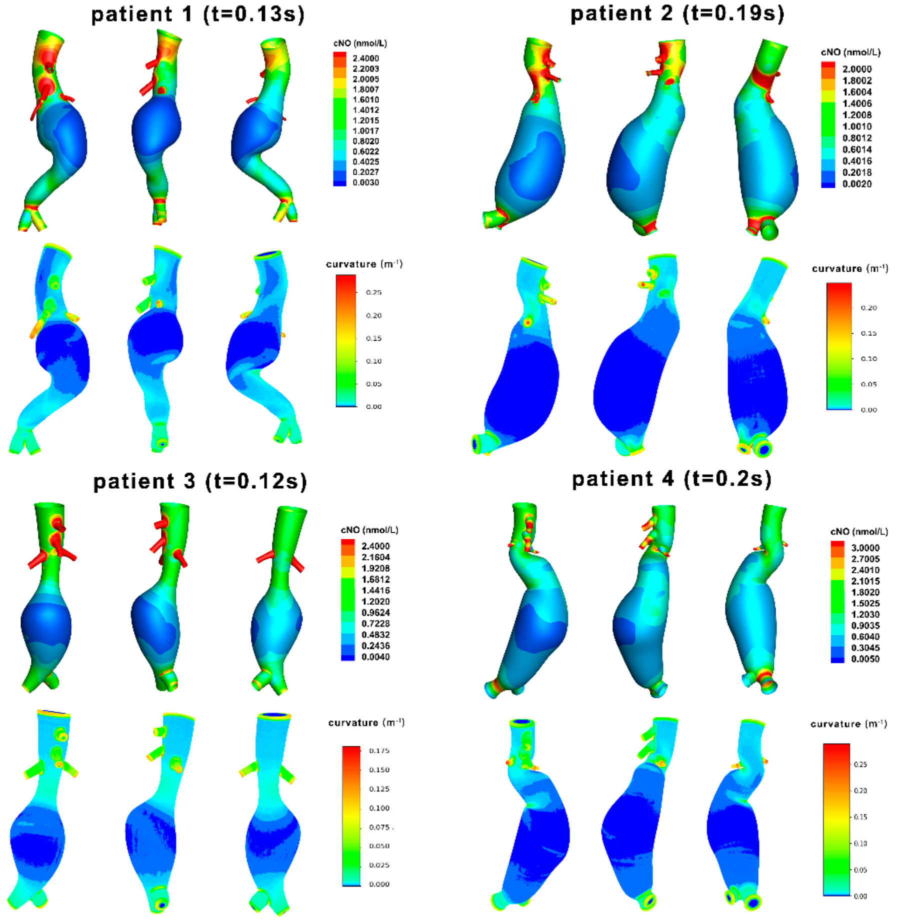

Figure S1. NO concentration at peak systole and vessel curvature.

Supplemental Table S1. Level of surface-averaged cNO and WSS in mesh independence check.

Patient 1	normal	fine	finer
number of elements	1365390	1512727	1915495
surface-averaged cNO			
plane-z- -0.4	0.026360581	0.026754172	0.026888311
plane-z- -0.38	0.012569943	0.012441647	0.012323546
plane-z- -0.37	0.014808814	0.01467892	0.014268914
plane-z- -0.33	0.005413057	0.005357725	0.005626744
plane-z- -0.32	0.003380169	0.003460418	0.003446627
surface-averaged wss	0.36888348	0.37343324	0.37363706
Patient 2			
number of elements	1971864	2137828	2469756
surface-averaged cNO			
plane-z- -0.46	0.0070863828	0.0070454116	0.0069753115
plane-z- -0.45	0.0039578721	0.0039158891	0.0038462939
plane-z- -0.44	0.0030943513	0.0030511535	0.0029801502
plane-z- -0.43	0.003286164	0.0032367388	0.0031542467
plane-z- -0.42	0.0038842359	0.0038296609	0.0037377982
plane-z- -0.41	0.0045278705	0.0044722875	0.004379082
plane-z- -0.40	0.0050822183	0.0050281286	0.0049381787
plane-z- -0.39	0.0053637467	0.0053145734	0.005232938
surface-averaged wss	0.10371065	0.10374334	0.10381287
Patient 3			

number of elements	1655741	2933659	3482527
surface-averaged cNO			
plane-z- 0.15	0.012123023	0.012093301	0.012108947
plane-z- 0.16	0.0052824356	0.0051696783	0.0051695613
plane-z- 0.17	0.0026208138	0.002565673	0.0025652562
plane-z- 0.18	0.0017626625	0.0017285208	0.0017280897
plane-z- 0.19	0.0015149706	0.0014870545	0.0014863859
plane-z- 0.20	0.0017228887	0.0016994206	0.0016988258
plane-z- 0.21	0.0029461604	0.0029134589	0.0029122006
surface-averaged wss	0.3219143	0.32260957	0.32278036
Patient 4			
number of elements	1174620	1300140	1832183
surface-averaged cNO			
plane-z- -0.4	0.0076936629	0.0077619175	0.007769854
plane-z- -0.39	0.0064646422	0.0066498844	0.0067289375
plane-z- -0.38	0.0069564211	0.0068544771	0.0071284115
plane-z- -0.37	0.0067511601	0.0066533192	0.0067350532
plane-z- -0.36	0.0053922184	0.0053276589	0.0053335552
plane-z- -0.35	0.0040723291	0.0039852309	0.0038950739
plane-z- -0.34	0.0034455513	0.0034063299	0.0033695381
plane-z- -0.33	0.0035986842	0.003552232	0.0034958484
surface-averaged wss	0.12708276	0.12744479	0.12801787

Supplemental Table S2. Percentage change of value in mesh independence check.

	**percentage change (normal-fine)**	**percentage change (fine-finer)**
Patient 1		
surface-averaged cNO		
plane-z- -0.4	1.493%	0.499%
plane-z- -0.38	1.021%	0.958%
plane-z- -0.37	0.877%	2.873%
plane-z- -0.33	2.738%	4.781%
plane-z- -0.32	1.022%	0.400%
surface-averaged wss	1.233%	0.055%
Patient 2		
surface-averaged cNO		
plane-z- -0.46	0.578%	0.995%
plane-z- -0.45	1.061%	1.777%
plane-z- -0.44	1.396%	2.327%
plane-z- -0.43	1.504%	2.549%
plane-z- -0.42	1.405%	2.399%
plane-z- -0.41	1.228%	2.084%
plane-z- -0.40	1.064%	1.789%
plane-z- -0.39	0.917%	1.536%
surface-averaged wss	0.032%	0.067%
Patient 3		
surface-averaged cNO		
plane-z- 0.15	0.245%	0.129%
plane-z- 0.16	2.135%	0.002%
plane-z- 0.17	2.104%	0.016%
plane-z- 0.18	1.937%	0.025%
plane-z- 0.19	1.843%	0.045%
plane-z- 0.20	1.362%	0.035%
plane-z- 0.21	1.110%	0.043%
surface-averaged wss	0.216%	0.0529%
Patient 4		
surface-averaged cNO		
plane-z- -0.4	0.887%	0.102%
plane-z- -0.39	2.865%	1.189%
plane-z- -0.38	1.465%	3.996%
plane-z- -0.37	1.449%	1.228%

plane-z- -0.36	1.197%	0.111%
plane-z- -0.35	2.139%	2.262%
plane-z- -0.34	1.138%	1.080%
plane-z- -0.33	1.291%	1.587%
surface-averaged wss	0.2849%	0.450%
